# Tough doughnuts: affect and the modulation of attention

**DOI:** 10.3389/fnhum.2013.00876

**Published:** 2013-12-18

**Authors:** Janani Dhinakaran, Maarten De Vos, Jeremy D. Thorne, Niclas Braun, Jolanda Janson, Cornelia Kranczioch

**Affiliations:** ^1^Neuropsychology Lab, Department of Psychology, European Medical School, Carl von Ossietzky University of OldenburgOldenburg, Germany; ^2^Methods in Neurocognitive Psychology Group, Department of Psychology, Carl von Ossietzky University of OldenburgOldenburg, Germany; ^3^Cluster of excellence “Hearing4all,” European Medical School, Carl von Ossietzky University of OldenburgOldenburg, Germany; ^4^Neurosensory Science Research Group, Carl von Ossietzky University of OldenburgOldenburg, Germany

**Keywords:** attentional modulation, affect, emotion, steady state visual evoked potential, rapid serial visual presentation, attention, EEG, visual attention

## Abstract

Positive affect has been associated with improvement in performance in various attentional domains. Negative affect has been associated with narrowing of attention and lowering of performance in attentional tasks. Previous behavioral studies have put forth the diffuse mental state idea as the mechanism of these effects, where attentional resources are more evenly distributed during positive affect and more focused during negative affect. To explore neural correlates of this mechanism, a two-stream rapid serial visual presentation (RSVP) paradigm with centrally presented, overlapping streams was used. Participants attended one of the streams at a time and steady-state visual evoked potentials (ssVEP) in response to the attended and unattended streams were recorded in a positive, negative or neutral affect state. We predicted that in the positive affect condition, ssVEP responses to the attended and the unattended stream would be more alike than in a neutral condition. In the negative affect condition, as an expression of a less diffuse mental state, ssVEP responses were predicted to be more dissimilar. Self-assessments confirmed the effectiveness of the emotional manipulation. In the negative affect condition power was found to be higher than in the neutral condition. However, the modulations in the ssVEP did not reflect the predicted neural correlate of the diffuse mental state mechanism. Thus, the results provide evidence for negative affect modulating attention but suggest that the diffuse mental state is not a spatially oriented phenomenon.

## Introduction

Human attention has been shown to be flexible within a range of capacity. This has been explored in studies of temporal attention, in which mood changes are induced, or secondary working memory tasks introduced while participants perform a temporal attention task (Dreisbach and Goschke, 2004; Olivers and Nieuwenhuis, 2005, 2006). Evidence shows that both the positive emotion manipulation and the task-irrelevant mental activity improved temporal attention (Olivers and Nieuwenhuis, 2005, 2006). Similar findings for the effect of emotionally potent positive stimuli have also been reported for other aspects of attention and cognitive domains such as visual neglect, overall breadth of attention, cognitive flexibility, conscious perception, early perception, sustained attention, sensory motor skills and spatial abilities (Thompson et al., 2001; Beilock et al., 2002; Dreisbach and Goschke, 2004; Phelps et al., 2006; Rowe et al., 2007; Schellenberg et al., 2007; Kuhbandner et al., 2009; Soto et al., 2009). Olivers and Nieuwenhuis were the first to suggest that the mechanism by which this enhancement may work is that of a “diffuse mental state.” They explain this with respect to what they coined the “overinvestment hypothesis” in which they suggest that if too much attentional resource is invested in a task, distractors are likely to be processed and this may in turn impede target detection. If attentional resources are instead diffused, either because of a secondary task that diverts attention from the primary task, or by the induction of positive affect, the threshold of attention would change to a level that would aid target detection because distractors would be ignored. In other words, the diffuse mental state, as caused by positive affect or a secondary task, would prevent over-processing of attended information and hence reduce the interference from distractors. Olivers and Nieuwenhuis refer to the effect of positive affect on attention as the “positive affect hypothesis” (Olivers and Nieuwenhuis, 2006). Though they do not offer a detailed definition of the term “diffuse,” they indicate that this state is characterized by a more even distribution of attentional resources.

Studies also show that the converse effect occurs such that negative affect is associated with a narrowing and focusing of cognitive processing (Fredrickson and Branigan, 2005; Storbeck and Clore, 2005; Bäuml and Kuhbandner, 2007; Gable and Harmon-Jones, 2010). Jefferies et al. (2008) focused on the experimental modulation of valence and arousal and its effect on the attentional blink (AB). The AB is a phenomenon of temporal attention, whereby the conscious perception of a second target is impaired when it appears within about 200–500 ms after a first target (Raymond et al., 1992). Jefferies et al. (2008) found that the AB was largest for participants in a state of high arousal combined with negative affect (anxious) and lowest for those in a state of low arousal combined with negative affect (sad). Participants with positive affect combined with either high or low arousal showed intermediate performance. Moreover, other studies show that those with severe dysphoria (an emotional state characterized by anxiety, depression or unease) tend to show longer and larger ABs (Rokke et al., 2002), and that negative disposition increases the AB magnitude (Maclean et al., 2010). Maclean et al. (2010) used the positive affect hypothesis as proposed by Olivers and Nieuwenhuis (2006) to interpret their findings and suggested that the performance differences they observed related to a more focused cognitive style in individuals with greater dispositional negative affect. The authors argued that this focused cognitive style causes these individuals to overinvest their attention to the rapid stream of non-targets, which then negatively affects the processing of the second target. To our knowledge, previous studies have not explored the neural correlates of the modulation of the focus of attention resulting from either positive or negative affect state.

Several physiological and behavioral studies have suggested that spatial location plays an important role in visual processing. This is often referred to by phrases such as “spotlight of attention,” “zoom lens” or “doughnut” (e.g., Eriksen and James, 1986; Posner and Petersen, 1990; Müller and Hübner, 2002). Among other findings, this idea is supported by the finding that in the electroencephalogram (EEG), visual evoked potentials (VEPs) elicited by stimuli presented inside the spotlight or inside the attended area have higher amplitudes than VEPs evoked by stimuli presented outside (Luck and Hillyard, 1994; Hillyard et al., 1998; Luck and Ford, 1998). Similar findings have been reported for the steady-state visual evoked potentials (ssVEP) (Müller and Hillyard, 2000; Müller and Hübner, 2002; Müller et al., 2003). The ssVEP can be evoked by flickering light, but also by a rapid serial visual presentation (RSVP) of simple or complex visual stimuli, and is seen as a robust and sensitive physiological measure of visual information processing (for a recent review, see Vialatte et al., 2010). Most relevant for the present study, it has been convincingly shown that ssVEP power reflects whether central visual information is attended or ignored (Müller and Hübner, 2002).

The diffuse mental state idea has to date been based on indirect, behavioral evidence (Olivers and Nieuwenhuis, 2006; Maclean et al., 2010). With the present study we therefore aimed to add more direct evidence by studying neural correlates of the diffuse mental state as a spatially-oriented phenomenon. We reasoned that the sensitivity of the ssVEP to attentional manipulations makes it ideally suited for this purpose. We hypothesized that if an experimental manipulation of affect results in a more diffuse mental state or a more focused state, this should be reflected in a modulation of the annular or doughnut shaped spotlight of attention, i.e., the ssVEP attention effect. The attention effect is the increase in ssVEP power if the evoking stimulus stream is attended. In detail, the inducing of positive affect should result in a more diffuse mental state, a spatially more even distribution of attention, and, in consequence, the use of less attentional resource to process a to-be-attended RSVP stream. This would be reflected in a smaller power difference between the ssVEPs evoked by the to-be-attended and the to-be-ignored RSVP streams in a positive affect condition as compared to a neutral affect condition. Following the converse of the positive affect hypothesis, we furthermore hypothesized that a negative affect manipulation would induce a less diffuse mental state and help the focusing of attentional resources on the to-be-attended RSVP stream. This would be reflected in a larger power difference between the ssVEPs evoked by the to-be-attended and the to-be-ignored RSVP streams in a negative affect condition as compared to a neutral affect condition. Finding such differences would substantiate the idea of a “diffuse mental state” and would add to our understanding of how affect states can influence attention.

## Methods

### Subjects

The aim was to have 15 participants in the negative affect and the positive affect groups. Allowing for elimination based on effectiveness of emotional manipulation or quality of the EEG data, we recorded a total of 39 right-handed subjects (24 females) between the ages of 19 and 33 (*M* = 23.9 years) who were monetarily compensated for their time. All had normal or corrected-to-normal vision and were free of past or current neurological or psychiatric disorders. All participants were right handed as assessed by the Edinburgh Handedness Inventory (Oldfield, 1971). Due to technical problems during recording of the EEG two participants had to be excluded from the sample. Five more subjects were excluded because they reported feeling the opposite effect of the intended emotional manipulation; for example, if they were in the positive group and the affect conditions made them feel more negative instead of more positive. The final sample consisted of 18 participants in the positive affect group (12 female, mean age 23.5, range 21–28 years), and 14 in the negative affect group (10 females, mean age 24.2, range 21–27 years). Beck Depression Inventory-II (Beck et al., 1996) scores gave no indication of the presence of clinical depression (*M* = 5.6, *SD* = 4.9). The study was run in accordance to the declaration of Helsinki and was approved by the ethics committee of the University of Oldenburg.

### Stimuli and procedure

#### Stimuli and setup

**Figure 1:**
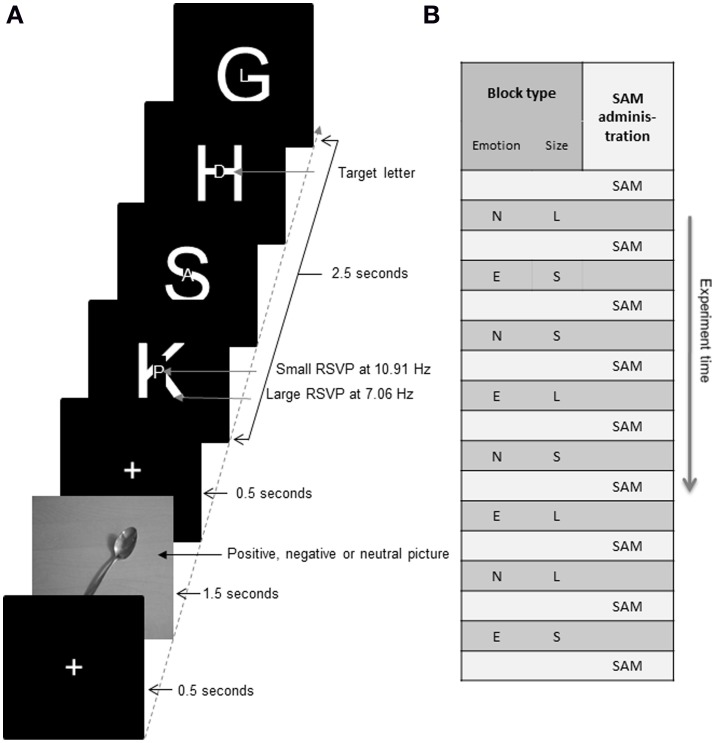
**(A)** Schematic illustration of the experimental paradigm that depicts the arrangement of the RSVP stimuli in terms of the time layout of a single trial. The duration of each section of the trial is indicated alongside. The picture of the spoon is courtesy of the authors and serves for illustration only. **(B)** Schematic layout of a possible block order. N and E indicate blocks where either neutral pictures (N), or emotion pictures (E being positive or negative pictures based on which group the participant was assigned to) were shown before each trial. L and S stand for whether the instructions asked the participant to attend the large or small letter stream. SAM scores were taken once before the experiment began and then again after each block.

A trial consisted of a picture taken from the international affective picture system (IAPS; Lang et al., 1999) and a two-stream RSVP of flickering, uppercase letters. The trial started with the presentation of a fixation cross for 500 ms, which was followed by an IAPS picture presented for 1500 ms. This was then followed by another 500 ms of the fixation cross and the RSVP streams of flickering letters that continued for 2500 ms. Thus, a whole trial lasted for 5 s (see Figure 1). All stimuli were presented on a 23.6 inch computer screen approximately 160 cm in front of the fixed chair in which the participants were seated. The computer screen was mounted outside the recording booth. Presentation Version 14.8 Build 12 30.10 (Neurobehavioral Systems, San Francisco, USA) was used to deliver the stimuli.

IAPS pictures fell into one of three categories: positive, negative, or neutral. For each category 20 pictures were chosen from the IAPS. Each picture has a standardized rating on a nine-point scale version of self-assessment-manikins (SAM) (Bradley and Lang, 1994) for three dimensions of inner state: valence (from positive to negative), arousal (excited to calm), and dominance (big to small). SAM ratings for the selected IAPS pictures had valence means of 7.5, 2.9, and 5.0 and arousal means of 4.6, 5.2, and 3.4 for the positive, negative, and neutral pictures, respectively. Dominance ratings were of no interest for the present study and are therefore not discussed in this paper. (Please note: there are several versions of SAM, apart from the nine-point version used for standardizing IAPS pictures. They include the modified-nine-point scale, five and seven point scale versions as well as all options with a flipped scale.) IAPS pictures were shown in color, and delimited a viewing angle of 10° × 7.5°. Within each experimental block, pictures were all of the same emotional category. Order of presentation was randomized both within each block and across participants.

Similar to the trials in the study by Müller and Hübner (2002) the trials consisted of two RSVP streams, one of large uppercase letters and the other of small uppercase letters within the larger letter presented at fixation. Letters as well as the fixation cross were presented white on black. The large letters that made up the first RSVP stream delimited a viewing angle of 2.11° × 1.94°. The second RSVP stream consisted of the smaller letters that subtended a viewing angle of 0.53° × 0.53°. All letters of the Latin alphabet were used for the RSVP streams except I, J, O, P, and W. Presentation frequencies of the two streams were synchronized to the 120 Hz refresh rate of the monitor. Each large letter was presented for 17 frames or 141.7 ms resulting in a frequency of 7.06 Hz. Each small letter was presented for 11 frames or 91.7 ms resulting in a frequency of 10.91 Hz. For the sake of convenience, we will henceforth refer to the two frequencies as simply 7 or 11 Hz.

#### Procedure

Before the start of the experiment subjects answered a set of questionnaires. These included our standard lab questionnaire inquiring about the current health state of the participant, the Edinburgh Handedness Inventory (Oldfield, 1971) and the Beck Depression Inventory (Beck et al., 1996).

After completing the questionnaires, the EEG recording was set up. Participants then completed a brief non-verbal self-report measure of their current emotional states, a three-dimension, flipped modified-nine-point scale version of the SAM which included valence, arousal and dominance to serve as a baseline for later measurements of the effectiveness of the emotional manipulation. Finally, general task instructions were given to the participants and an example neutral trial was shown on the screen. Participants were encouraged to ask if they had any questions. If they had no further questions the experiment started with the first block-specific instruction which asked participants to fixate on the center of the screen, and to attend to the large or small letter stream.

In each block there were 72 trials, resulting in a block duration of about 6 min. As illustrated in Figure 1B, the experiment consisted of eight blocks, with alternating neutral and emotion condition blocks. Half of the subjects began with the neutral and the other half of subjects began with the emotional condition. The emotion condition would be either positive or negative based on which group the participant was randomly assigned to. Whether participants started with the “attend large” or the “attend small” instruction was counterbalanced across participants. The order of instructions within an experiment followed an ABBABAAB design. That is, if a participant started with the instruction “attend large” the order of instructions was LSSLSLLS, while starting with the instruction “attend small” was linked to the order SLLSLSSL, where L denotes attending the large letter stream and S denotes attending the small letter stream. Combining the alternation of emotion and neutral conditions with the two size orders resulted in four different orders in which the blocks could be presented to the participants. As an example, block order could be L/Neutral–S/Emotion–S/Neutral–L/Emotion–S/Neutral–L/Emotion–L/Neutral–S/Emotion. After each block, participants filled in the SAM again and were given a short break.

To ensure that participants paid attention to the instructed RSVP stream a behavioral task was introduced. Participants were told to press a button as quickly as possible if they detected the letter H in the to-be-attended RSVP stream. The button was mounted on a custom-made response pad that lay on a table in front of the participant and was to be pressed with the right index finger. The letter H could occur from 700 to 2125 ms after the onset of the RSVP streams. Within a given trial, the target letter H occurred up to two times either in the small or the large RSVP stream, but never in both. In cases of two target letters, Hs were separated by 990 ms so as to completely avoid any interference from the AB phenomenon (Raymond et al., 1992).

### Physiological recordings

EEG was recorded from a 94-channel equidistant Ag-AgCl electrode cap (EASYCAP, Herrsching, Germany). One additional electrode was mounted below the right eye to monitor eye movements. An electrode placed on the nose tip served as online-reference. Data were recorded using BrainVision recorder (Version 1.10), together with Brainamp DC Amplifiers (Brain Products GmbH, Gilching, Germany). Electrode impedances were kept below 8 kΩ before data acquisition. Resolution was 0.1 μV with a range of ±3.28 mV. Data were recorded with a sampling rate of 1000 Hz with filter settings of 0.016 Hz for the high pass filter and 250 Hz for the low pass filter.

### Data processing

#### Behavioral data

Hits and false alarms were calculated for the target detection task. SAM ratings were extracted for the dimensions arousal and valence.

#### EEG

EEG data were analyzed using EEGLAB v9.0.4.4b (Delorme and Makeig, 2004), an open source MATLAB R2010b (The MathWorks Inc., Natick, MA, USA) based software, and custom Matlab-based scripts. The EEG was filtered offline with a low pass filter of 100 Hz, resampled to 250 Hz, and passed through a high pass filter of 1 Hz. In a two-pass procedure, dummy events were first created and the continuous data set epoched into segments of 5000 ms duration. According to lab procedures (see De Vos et al., 2012), principal components analysis (PCA) was run prior to ICA to reduce the dimensionality of the data, and ICA was then based on 45 principal components. Extended infomax Independent Component Analysis (ICA) was run (Bell and Sejnowski, 1995; Lee et al., 1999) and ICA weights obtained from this were stored for later use. In the second pass, the original data of the experiment were reloaded and then filtered with a low pass filter of 100 Hz, resampled to 250 Hz, and passed through a high pass filter of 0.1 Hz. ICA weights from the first pass were then imported. ICs were examined individually in the epoched data set and ICs reflecting stereotypical artifacts such as eye blinks, heartbeats, and lateral eye movements were removed. After ICA-based data pruning, an automatic rejection algorithm was run to identify and remove any strange epochs using joint probability of recorded electrodes at each time point and visual inspection.

The cleaned EEG epochs were then split based on condition. To examine the effect of attention at each frequency, a fast Fourier transform (FFT) was run on the averaged trials of these condition sets to extract power values for each channel at the two frequencies at which the letters were presented. In order to avoid the visual evoked response to flicker onset, the first 500 ms of each trial was excluded from this analysis. Grand mean averages of the power values were used to make topographical maps of the neutral condition. Based on visual inspection of peak ssVEP topography, a region of interest was defined in the posterior region, which included eight parieto-occipital channels, four in each hemisphere. These roughly corresponded to the region occupied by PO7, PO3, O1 and the analogous channels PO4, PO8, and O2 in the 10-10 system. Power values from these channels were extracted for statistical analysis.

### Statistical analysis

Firstly, to determine whether there was an impact of the emotion manipulation, SAM data was tested for a significant difference between the neutral and emotion conditions in each group.

D prime (d′) was calculated using the Z-transformed values of the proportion of hits and false alarms. D′ is a statistical measure of the difference between hits and false alarms and indicates sensitivity in a detection task. The larger the d′ value, the more sensitive participants are to the difference between signal present and signal absent distributions. The behavioral task served only to ensure attention on the instructed stream, and it varied considerably from standard AB tasks in which behavioral effects have been previously observed. Therefore, no hypothesis was made regarding this data. However, a two-way ANOVA was run on these values with affect condition (neutral, emotion) and attended letter stream (large, small) as factors, for each group separately, to test whether performance was affected by either the affect manipulation or the attention condition.

To test the main hypotheses, a three-way repeated measures ANOVA was run on the log EEG power values (Gasser et al., 1982) of each group with the frequency measured [7 Hz (slow and large RSVP stream), 11 Hz (fast and small RSVP stream)], the attended letter stream (7 Hz, 11 Hz) and the affect condition (emotion, neutral) as factors. If present, interactions were followed by *t-tests*. Mauchly's sphericity assumption was met for all reported values.

## Results

### SAM

Table 1 shows the mean SAM valence and arousal values and respective standard error of the mean in each group according to emotion condition. An independent-samples *t*-test was run to explore pre-existing group differences in valence and arousal values. No significant group differences were found. Paired *t*-tests were performed to compare the emotion and neutral conditions in each group. A significant effect of the emotional manipulation found in both groups confirmed its effectiveness: the positive group had a significantly lower mean valence during the emotion conditions than during the neutral conditions [*t*_(17)_ = −2.13, *p* < 0.05] and the negative group had a significantly higher mean valence during the emotion conditions compared to the neutral conditions [*t*_(13)_ = 2.25, *p* < 0.05]. Please note: the scoring of the flipped modified-nine-point scale version of SAM used here decreased with positive valence and increased with negative valence. There was no significant difference between the arousal ratings of the neutral or emotion conditions of either group.

**Table 1:** **SAM Scores for both groups in both affect conditions**.

**Group**	Positive groupvalence^*^	**SEM**	Negative group valence^*^	**SEM**	**Positive group arousal**	**SEM**	**Negative group arousal**	**SEM**
Neutral condition	5.0	0.25	4.5	0.27	5.5	0.25	5.4	0.27
Emotion condition	4.7	0.29	5.0	0.27	5.4	0.29	5.4	0.27

* Group difference was significant p < 0.05. Note. Lower scores in the valence scale indicated more positive affect.

### Behavior

Considering that 69% accuracy would be indicated by a d′ of 1.0, the mean d′ values on this task clearly indicated that participants paid attention to the stream they were instructed to attend. This was reflected by adequate performance of the target detection task in all conditions in both groups (Positive group: Emotion condition 2.3 and Neutral condition 2.24; Negative group: Emotion condition 2.16 and Neutral condition 2.18). A two-way ANOVA with affect condition (neutral, emotion) and attended letter stream (large, small) as factors was run to analyse the data further. In the positive group, a main effect of attended letter stream was found [*F*_(1, 17)_ = 12.69, *p* < 0.01] where response accuracy was significantly better in the large letter stream compared to the small letter stream (2.54 vs. 2.01). No other effects or interactions were seen in either group.

### EEG

**Figure 2:**
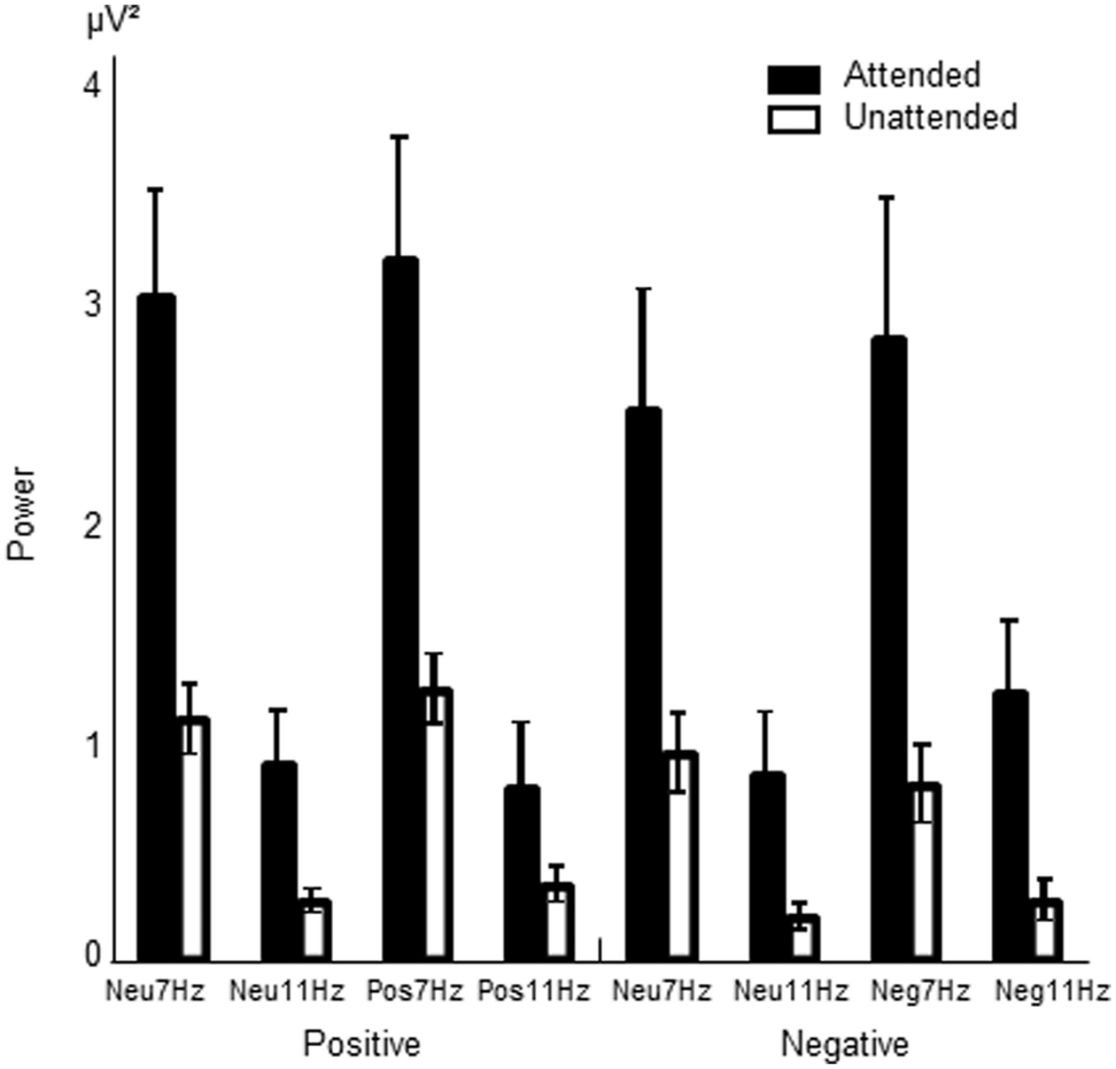
**Mean power values of the ssVEP and their respective standard errors of the mean (s.e.m) illustrating the attention effect.** Black bars show power values at the labeled frequency (7 or 11 Hz) when attended; white bars show power when unattended. Illustrated are the neutral and emotion conditions of the positive and negative groups.

As illustrated in Figure 2, both RSVP streams evoked frequency specific responses that were modulated by attention in that they were enhanced when the RSVP stream of corresponding frequency was attended. A three-way ANOVA, with power of the measured frequencies as the dependent variable and affect condition (positive or negative - depending on the group - and neutral), attended stream of letters (large, small) and measured frequency (7 Hz, 11 Hz) as factors, was performed for each group separately. Of particular interest was a three-way interaction between affect condition, attended stream of letters and measured frequency, which would indicate that emotion modulates the attention effect in the doughnut model of attention proposed by Müller and Hübner (2002).

#### Analysis in positive group

***Presence of attention effect.*** As expected, in the positive group the ANOVA showed a main effect of the measured frequency where the log power of the 7 Hz response was higher than that of the 11 Hz response (0.13 μV^2^ vs. −0.57 μV^2^; [*F*_(1, 17)_ = 63.37, *p* < 0.0001]), as well as an interaction between the attended letter stream and the measured frequency [*F*_(1, 17)_ = 42.9, *p* < 0.0001]. *t*-tests revealed that the log power of the 7 Hz response when attending the 7 Hz stream of large letters was significantly higher than the 7 Hz response when attending the 11 Hz stream of small letters [0.35 μV^2^ vs. −0.98 μV^2^; *t*_(17)_ = 6.90, *p* < 0.0001]. Similarly, the log power of the 11 Hz response when attending the 11 Hz stream of small letters was significantly higher than the 11 Hz response when attending the 7 Hz stream of large letters [−0.34 μV^2^ vs. −0.80 μV^2^; *t*_(17)_ = 4.60, *p* < 0.0001]. This shows the expected increase of power when a stream is attended as opposed to ignored.

***Presence of effect of affect.*** Contrary to our hypothesis, a three-way interaction was not observed [*F*_(1, 17)_ = 0.045, *p* = 0.83]. An interaction between affect condition and measured frequency and the main effect of affect condition were also absent.

#### Analysis in negative group

***Presence of attention effect.*** A similar analysis of the negative group showed the same effects as earlier mentioned in the positive group, that is, a main effect of measured frequency [7 Hz 0.003 μV^2^ vs. 11 Hz −0.61 μV^2^; *F*_(1, 13)_ = 61.94, *p* < 0.0001] as well as the interaction observed between attended stream and frequency measured [*F*_(1, 13)_ = 60.90, *p* < 0.0001]. *t*-tests revealed that the log power of the 7 Hz response when attending the 7 Hz stream of large letters was significantly higher than the 7 Hz response when attending the 11 Hz stream of small letters [0.26 μV^2^ vs. −0.25 μV^2^; *t*_(13)_ = 6.6, *p* < 0.0001]. Similarly, the log power of the 11 Hz response when attending the 11 Hz stream of small letters was significantly higher than the 11 Hz response when attending the 7 Hz stream of large letters [−0.31 μV^2^ vs. −0.91 μV^2^; *t*_(13)_ = 7.59, *p* < 0.0001]. Just as in the positive group, this shows the expected increase of power when a stream is attended as opposed to ignored.

**Figure 3:**
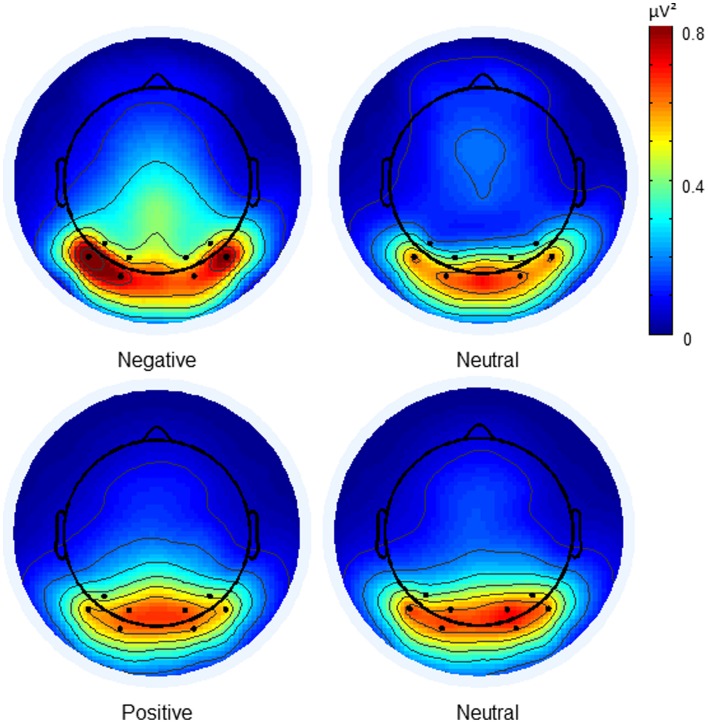
**Topographical maps of mean power values of the negative group during negative and neutral conditions.** The dots indicate the electrode locations (i.e., the region of interest) chosen for statistical analysis. Power is higher in the negative condition than in its neutral condition. The positive condition and its respective neutral condition are shown for comparison.

***Presence of effect of affect.*** Contrary to our hypothesis, the three-way interaction between affect condition (negative, neutral), attended stream of letters (large, small) and measured frequency (7 Hz, 11 Hz) was again not observed [*F*_(1, 13)_ = 0.068, *p* = 0.8]. However, a main effect of affect condition was seen where the mean power of the negative condition was significantly higher than that of the neutral condition [−0.28 μV^2^ vs. −0.33 μV^2^; *F*_(1, 13)_ = 4.66, *p* = 0.05]. This is illustrated in Figure 3. A significant interaction between the measured frequency and affect condition was also observed [*F*_(1, 13)_ = 6.01, *p* < 0.05]. When this was followed up by *t*-tests, it was found that the 11 Hz response was significantly higher in the negative condition than in the neutral condition [−0.55 μV^2^ vs. −0.67 μV^2^; *t*_(13)_ = −2.912, *p* < 0.05]. No equivalent difference was seen in the 7 Hz response [*t*_(13)_ = 0.61 *p* = 0.555].

## Discussion

The goal of this study was to investigate the neural correlates of the diffuse mental state idea proposed by Olivers and Nieuwenhuis (2006) as a spatially-oriented phenomenon. To this end, a two-stream RSVP paradigm was used (Müller and Hübner, 2002) in combination with an emotion manipulation. The attentional resources directed at processing the RSVP streams were measured by means of ssVEPs. The emotion manipulation was carried out between groups, with one group being exposed to positive and neutral pictures, the other group to negative and neutral pictures. We expected that if positive affect results in a more diffuse mental state, this would result in a more even distribution of attentional resources. This should be reflected in a reduction of the normally observed ssVEP attention effect, that is, the power increase seen when a stimulus stream is attended as compared to when it is ignored. Conversely, we expected that when negative affect focuses the attentional resources, this should be reflected in a corresponding enhancement in the ssVEP attention effect.

The basic paradigm was successful in that the attention effect was observed in both groups. Our EEG findings clearly replicate the finding of Müller and Hübner (2002) in that attention to one of two simultaneously presented RSVP streams resulted in an increase in power in the frequency corresponding to the attended stream's presentation rate. Performance data also confirm that participants attended the correct stream. It is important to note here, that the behavioral task was used for the verification of attention. It was not expected to be sensitive to the emotional manipulation. However, the changes in the SAM valence ratings clearly indicate that the emotional manipulation succeeded.

In the negative group, a main effect of affect was seen in which the power of the ssVEP in the negative condition was higher than that of the neutral condition. An interaction between measured frequency and affect condition was also seen whereby the increase in power observed in the negative condition was particularly strong in the 11 Hz response but absent in the 7 Hz response. This can be interpreted as an indication that negative affect had a task-independent, narrowing effect on the focus of attention. This is in line with the idea that negative affect narrows the mental state (Fenske and Eastwood, 2003; Smilek et al., 2006; Maclean et al., 2010).

While we see a reflection of the emotional manipulation in the negative group, a similar effect is not observed in the positive group. Perhaps this is because the emotional manipulation is not strong enough to reflect clearly in the physiology. IAPS pictures were chosen as a method of manipulating the mood, as they have been used widely and have effectively manipulated moods and performances in earlier studies (Dreisbach and Goschke, 2004; Olivers and Nieuwenhuis, 2006), however, other methods for manipulating mood might be more effective in inducing a positive affect state. For instance, it has been found that music positively affects performance on several tasks and tests, and also temporarily eliminates spatial neglect (Thompson et al., 2001; Olivers and Nieuwenhuis, 2005; Rowe et al., 2007; Graham et al., 2009; Soto et al., 2009). Thus, future research could investigate whether the ssVEP attention effect can be modulated if positive affect is induced by other methods than IAPS pictures.

In addition to the above idea, Schupp et al. (2007) showed that emotional attentional capture is reduced by the competing processing demands of a concurrent attentionally-demanding task. In the present study a related mechanism might have influenced the results. That is, the combination of attending either the small or the large letter stream while ignoring the other and looking for a target letter may have increased the attention demand to a level that similarly diminished the effect of the positive pictures.

With respect to the expected modulations based on the diffuse mental state idea, we did not observe the relative reduction in the ssVEP attention effect compared to the neutral condition in the positive condition, or the relative increase in attention effect in the negative condition. Although it remains possible that this result was due to a weak emotion manipulation, the significant main effect of SAM valence scores in both groups argues against this. Our experimental design was based upon the interpretation of the diffuse mental state as being a spatially-oriented phenomenon. We therefore expected that with the diffuse mental state as induced by positive affect, the spotlight of attention would expand spatially, and with negative affect causing a narrowing and focusing of attention, the spotlight would narrow. Our null result therefore suggests that the spatial “doughnut” model is tough and inflexible. However, another interpretation of this state is possible. Maclean et al. (2010) refer to the diffuse mental state in connection with increased cognitive flexibility and control that comes from more open and positive dispositions, implying that it can be seen more as an enhancer of more efficient resource allocation. Thus, we do not discount the principle of a diffuse mental state per se. Instead we suggest that an interpretation of such a state beyond a merely spatial phenomenon may be appropriate, although this remains to be directly tested.

In conclusion, this study provides further evidence for the modulation of attention by negative emotion. However, there was no evidence of a neural correlate of the diffuse mental state in a spatial conception. Future studies could try alternative tasks with less emphasis on the spatial component, as these may be better able to reflect a more, or less, diffuse mental state. Further studies may also benefit from a stronger affect manipulation, in particular for the induction of the positive affect state.

## Author contributions

Janani Dhinakaran and Cornelia Kranczioch developed the study concept and design. Testing, data collection and analysis were performed by Janani Dhinakaran. Cornelia Kranczioch, Maarten De Vos, Jolanda Janson, and Niclas Braun were involved in data analysis. Jeremy D. Thorne significantly supported programming of the experiment. Janani Dhinakaran drafted the paper with the support of Cornelia Kranczioch and critical revisions of Maarten De Vos, Jolanda Janson, Niclas Braun, Jeremy D. Thorne. All authors approved the final version of the paper for submission.

### Conflict of interest statement

The authors declare that the research was conducted in the absence of any commercial or financial relationships that could be construed as a potential conflict of interest.
